# Intramolecular Fuzzy Interactions Involving Intrinsically Disordered Domains

**DOI:** 10.3389/fmolb.2018.00039

**Published:** 2018-04-30

**Authors:** Miguel Arbesú, Guillermo Iruela, Héctor Fuentes, João M. C. Teixeira, Miquel Pons

**Affiliations:** BioNMR Laboratory, Inorganic and Organic Chemistry Department, University of Barcelona, Barcelona, Spain

**Keywords:** fuzzy complexes, intrinsically disordered proteins, Src family kinases, allostery, fuzzy domains, paramagnetic relaxation enhancement

## Abstract

Structural disorder is an essential ingredient for function in many proteins and protein complexes. Fuzzy complexes describe the many instances where disorder is maintained as a critical element of protein interactions. In this minireview we discuss how intramolecular fuzzy interactions function in signaling complexes. Focussing on the Src family of kinases, we argue that the intrinsically disordered domains that are unique for each of the family members and display a clear fingerprint of long range interactions in Src, might have critical roles as functional sensor or effectors and mediate allosteric communication via fuzzy interactions.

A large majority of proteins are build from domains, classically defined as functional autonomous folding units. In a typical divide-and-conquer approach, the structure-function analysis proceeds through the characterization of the individual domains followed by the study of their mutual interactions. This approach makes a clear distinction between the “functional” domains and the linkers separating them.

The same strategy is taken in the analysis of the also very abundant multiprotein complexes, in which the individual proteins are considered the building blocks (equivalent to domains of multidomain proteins). Key components of multiprotein complexes are scaffolding proteins, which would play the role of linkers in multidomain proteins.

The current view of protein-protein interactions is quite dynamic and intrinsically disordered regions (IDR) are increasingly recognized as key players.

In this minireview we shall summarize some important aspects of (intermolecular) protein binding by disordered proteins and extend them to the case of interdomain (i.e., intramolecular) binding using the c-Src family of kinases as an example.

## Protein interactions by intrinsically disordered proteins

Intrinsically disordered proteins (IDP) or proteins with long IDR form a significant portion of the proteome of eukaryotes and are specially prevalent in signaling and regulation complexes (Iakoucheva et al., 2002).

Protein complexes involving IDRs span a wide range of affinities and lifetimes as well as specificities (Tompa et al., 2015). A recent analysis of K_d_ value statistics in the curated DIBS database of IDR-folded protein complexes (Schad et al., 2017) confirms a wide range of affinities spanning from the subnanomolar to the milimolar regimes.

In their review on experimental thermodynamic data from binary protein complexes involving IDPs or ordered proteins, Teilum et al. (2015) found that the ΔG° values of their data sets involving IDPs were on average only 2.5 kcal mol^−1^ less stable than the values from complexes between ordered proteins. In the two sets, isothermal enthalpy-entropy compensation was observed, a general phenomenon in biomolecular recognition processes (Chodera and Mobley, 2013). Thus, favorable binding enthalpy is associated to a loss of entropy due to massive reduction of structural freedom degrees.

The interaction surfaces involving folded proteins or IDRs showed similar amino acid composition and size and the distribution of ΔH° values were statistically equal; so, all the destabilizing contribution had an entropic origin (–TΔS° > 0).

An entropic cost for binding an IDR is intuitively expected, however, the surprising result is its relatively small value suggesting compensatory mechanisms are an important component of IDR interactions. The importance of entropy compensation is highlighted by the similarity in the distribution of ΔH° values, a very interesting result in itself that emphasizes the underlying short-range similarities between protein-protein interactions involving folded and disordered proteins.

Flock et al. (2014) have reviewed the importance of entropy control to tune IDR function. The importance of entropy in the formation of complexes endows IDR-involving complexes with their unique functional characteristics as molecular rheostats and signal integrators, able to respond in a precise, continuous and dynamic way to varying combinations of inputs with specific outputs.

The formation of a stable complex between two proteins (i.e., with a negative ΔG° = ΔH° –T ΔS°) can be achieved by optimizing the enthalpy gain (ΔH° < 0), increasing the entropic gain (–T ΔS° < 0 = > ΔS° > 0) or minimizing the entropic loss (–T ΔS° ≈ 0). A fundamental aspect of the interplay between enthalpy and entropy components is their “locality.”

Enthalpy effects usually reflect local short-range interactions and may be considered additive and proportional to the contact surface. Thus, large enthalpy components are usually associated to large contact surfaces, although these interfaces do not have to be necessarily continuous. Electrostatic contributions to the enthalpy, however, are long range. They often drive the partners together (thus reducing the translational and possibly rotational entropy of the system) and, in the formed complex, enable dynamic interactions that minimize the entropy loss upon complex formation. A recent example of a picomolar interaction between two IDPs without a significant loss of flexibility is driven by electrostatics (Borgia et al., 2018).

## Interacting elements and multivalency

Entropy contains “local” components associated to the degree of structure achieved by the contact regions of the two interacting partners, as well as more global contributions of which we may distinguish (i) the effect of regions that can remain highly flexible in the complex (thus not contributing an entropic penalty to binding), (ii) the possible preexistence of long range intramolecular contacts restricting the conformational freedom in the free IDR (therefore minimizing the loss of entropy upon complex formation), and (iii) the configurational entropy arising from multiple alternative binding poses (“microstates”) contributing to the bound state.

The first two situations reduce the entropic cost of binding through IDRs and correspond to the strategies of not to pay (i) or pre-pay (ii). The third situation actually contributes an entropic gain.

In the interacting regions, pre-pay strategies may take the form of preformed structural elements retained in the complex (Davey et al., 2012; Pancsa and Fuxreiter, 2012) or bound solvent molecules that are retained in the complex in water-mediated interactions (London et al., 2010).

The dominant role of entropy in protein interactions is not restricted to IDPs. The changes in internal dynamics of the catabolite activator protein (CAP), measured by NMR in the entirely protein, explain the dramatic changes in affinity observed in CAP variants that form complexes with identical interfaces (Tzeng and Kalodimos, 2012).

In IDRs the change in entropy upon binding is determined by the interplay between local and global effects. Short linear motifs (LM) play an important role in IDR interfaces. Although the definition of LM is based on bioinformatic studies, they can be interpreted using structural and dynamic concepts. LM are often formed by hydrophobic residues grafted onto a maleable template (Fuxreiter et al., 2007). The expected lower enthalpy of the interaction by short elements, as compared to the large rigid interfaces between ordered proteins, can be partially compensated by the fact that IDPs often adopt extended conformations permitting short motifs to establish a variety of interactions through virtually any element of their backbone or side chains, thus their interacting interfaces have a larger effective area per residue than those of ordered proteins (Gunasekaran et al., 2003). In addition, these short stretches are modular recognition elements that can be combined to form multivalent complexes. Thus, a favorable binding enthalpy, comparable to that found associated to a large, rigid interface, can be achieved by weaker but multiple sparse anchoring elements (Cumberworth et al., 2013). The participating groups may be difficult to identify either experimentally, because interactions are weak, or statistically, because they may appear in a variety of combinations that are not repeated “motifs” (Van Roey et al., 2014).

The number and intrinsic properties of individual interacting regions, as well as the size and dynamic properties of the spacers between them collectively, and therefore non-linearly, determine the binding properties of IDRs.

The effects of multivalent binding have been recognized for a long time, beyond the field of IDPs. The strength by which a multivalent antibiotic binds to its antigen, termed avidity (Crothers and Metzger, 1972), can be explained by the fact that when one of the sites is bound to its cognate site receptor, a second site located close-by binds cooperatively, basically because of the lower entropic cost of a (pseudo)-intramolecular interaction (Kitov and Bundle, 2003). If the linker connecting the two sites is flexible, the average distance between the sites is the main factor determining the cooperativity. If the flexibility is limited, the linker may also modulate the relative orientation of the components of the second interacting site. A consequence of the model is that avidity may be modulated either by modifying the interacting sites or the flexibility of the linker (Cerofolini et al., 2013).

The avidity model assumes multiple binding sites but the interacting partners for each site are not interchangeable. The scenario in which the multiple interaction sites in one of the molecules can interact with, and therefore compete for, the same site of the second molecule is referred to as **allovalency** (Klein et al., 2003). Allovalency predicts a dependency on the number of interacting sites, not through simultaneous cooperative binding, because all of them compete for a single site in the second molecule, but through local concentration and rebinding. In the defining example, the binding of Sic1 to Cdc4, the number of interacting sites is actively modulated by the random phosphorylation of up to ten serine and threonine residues, showing a sharp increase in the fraction of bound form after six of them are phosphorylated (Mittag et al., 2010).

## Fuzzy complexes and multivalency

**Fuzzy complexes**, introduced by Tompa and Fuxreiter (2008) describe binding situations in which at least one of the elements in the complex remains dynamic. Therefore, the complex cannot be properly described by a defined structure but has the characteristics of an heterogeneous ensemble. Importantly, the interaction heterogeneity of the fuzzy complex is an essential component of the functional outcome of complex formation. The functional character of the retained disorder, thus, differentiates a fuzzy complex from a complex including a random region in non-specific contact with the partner. An expanded repertoire of examples can be found in recent reviews (Fuxreiter, 2012; Fuxreiter and Tompa, 2012; Sharma et al., 2015; Miskei et al., 2017).

Structural disorder in fuzzy complexes represents a continuum, from rather rigid polymorphic complexes displaying static disorder with only a few alternative conformations to highly dynamic random complexes. The proportion between regions directly involved in short range contacts and connector regions decreases in this series. Individual regions contributing to ΔH° < 0 become smaller but may increase in number, thus a favorable enthalpy contribution can be retained. Splitting the interaction interface in many smaller areas, each binding weakly and with high promiscuity, enhances binding degeneracy that contributes an additional entropic term, Δ${\text{S}}_{\text{configurational}}^{{}^{\circ}}$, which reflects the contribution stemming from the different forms in which the IDR and its partner can associate.

A recent experimental example is the detailed study of the thermodynamics of the fuzzy complex between the C-terminal IDR of antitoxin CcdA, which adopts α helical structure at the time of binding the toxin dimer CcdB (HadŽi et al., 2017). The authors perform a series of mutations that affect contacting and non-contacting residues. Their results show that mutations in residues not directly involved in protein:protein interaction reduce the degree of structuration both in the bound and free forms (ΔΔ${\text{S}}_{\text{conformational}}^{{}^{\circ}}$ ≈ 0), but also promote alternative isoenergetic configurations (ΔΔ${\text{S}}_{\text{configurational}}^{{}^{\circ}}$ > 0 and ΔΔH° ≈ 0) thus minimizing the particular ΔG° of the mutant complex.

## NMR fingerprint of long-range organization of IDR

Fuzziness is not associated to promiscuous binding. The selectivity is encoded in the dynamic, non random, organization of distant potentially interacting regions.

Operationally, a very efficient method to map a set of long range interactions is by measuring the paramagnetic relaxation enhancement (PRE) along the sequence induced by one or several paramagnetic tags (Clore and Iwahara, 2009). Since paramagnetic effects are sensitive to transient interactions and efficient over considerable distances, in the case of disordered proteins the key aspect is to differentiate specific from random coil effects. In this respect, the Konrat's group have introduced the concept of paramagnetic relaxation interference (PRI) by comparing the simultaneous effect of two paramagnetic centers (Kurzbach et al., 2016) with the sum of the individual effects. A differences between these values requires that the two sites move in a correlated fashion. An alternative, often simpler, approach is to compare the observed PREs with the predictions of a random coil model. The ΔPRE analysis (Arbesú et al., 2017) clearly identifies the relevant transient contacts in IDRs.

ΔPRE analysis of several paramagnetically tagged forms of the intrinsically disordered N-terminus of c-Src in the presence and in the absence of the folded neighboring SH3 domain show a very similar profile, confirming the presence of a conserved set of non-random long-range interactions and validating the use of the term **domain** for this intrinsically disordered region (Arbesú et al., 2017). Subtitution of residues important for IDR pre-organization and interdomain contacts showed that the ΔPRE profiles are generally conserved upon different perturbations—e.g., same profile trends, location of maxima and minima, etc.—but can also reflect the functional loss of interactions—i.e., consistent contact reduction upon substitution. ΔPRE profiling thus provides a structural signature that captures non random ensemble conformational preferences and their associated dynamics. This simple method enables facile comparison for carrying out functional analysis based on mutations.

In an analysis of the Pfam database, which identifies domains based on multiple sequence alignments, Tompa et al. (2009) found that a substantial number of the sequence defined domains contained disordered regions and confirmed that disordered domains are inheritable, evolvable, and functional units. Some domains, such as the Unique domain, which is the most discriminating feature of the distinct Src family kinases (SFK), is not identified as a domain using multisequence alignment methods. This is not surprising since sequence variability is its defining characteristic. We argue that the requirement of “autonomous folding,” which would identify a domain without using sequence conservation data, could be replaced in the field of IDPs by that of a conserved, non-random, set of long range interactions.

The PRE and chemical shift perturbation analysis of wild-type Src as well as a number of mutated or truncated variants, also showed the interaction between the intrinsically disordered domain and specific regions of the SH3 domain. Interestingly, the most affected regions in the folded scaffold could be mapped to the loops that decorate its surface. The interaction between the disordered N-terminal region of c-Src and the SH3 domain has the characteristics associated to fuzzy complexes: (i) the disordered region remains highly dynamic, as seen by NMR, (ii) its overall dimension is affected by the presence of the SH3 domain, as seen by Small Angle X-ray Scattering, (iii) the local perturbations sensed by chemical shifts are affected by modifications in distant, well defined parts of the protein, and (iv) mutations in the disordered region cause strong functional effects in the entire protein.

## Intramolecular fuzzy complexes as signal sensors

**Figure 1:**
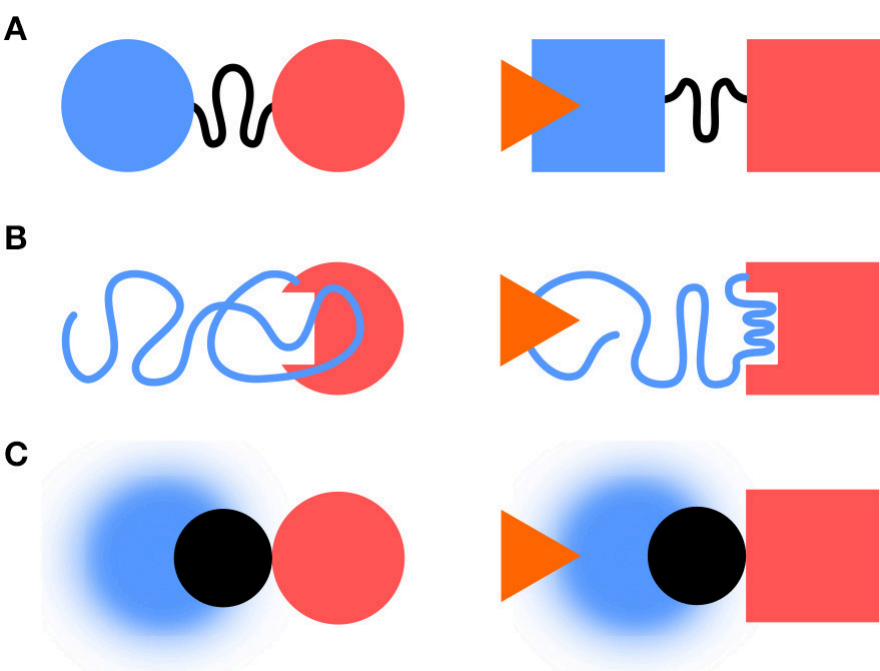
IDRs and information transfer. IDRs can act as linkers, effectors, or sensors. **(A)** In the linker case, the IDR transmits the information between folded sensor and an effector domains. **(B)** The IDR can become an effector, e.g., by folding as a response to a stimulus. **(C)** A fuzzy complex, like in Src, can act as a sensor with the folded SH3 domain (in black) taking the role of the linker.

Classical descriptions of multidomain signaling proteins distinguish between regulatory/sensor and catalytical/effector domains. IDR can act as linkers, effectors or sensors (Figure 1). IDR-mediated signaling enables complex regulatory behavior, including multiple signal integration and rheostat-like graded responses (Tompa, 2014).

IDRs can act as linkers through which information is propagated to distant regions. This can occur without concomitant structuration through remodeling of the protein free energy landscape affecting the conformer populations and causing specific functional outputs (Tsai et al., 1999; Hilser and Thompson, 2007; Ma et al., 2011; Montlagh et al., 2014). Examples include include the DNA binding Ets-1 transcription factor (Pufall, 2005), the Sic1 cell cycle protein (Mittag et al., 2008), or the Drosophila Ultrabithorax transcription factor (Liu et al., 2008).

IDR conformational ensembles can be modified by “external” signals and modulated by “internal” parameters, such as post-translational modifications. Thus, they can act as sensitive **sensors** with tunable selectivity and sensitivity. In a recent work, the effect of a small drug interacting with a disordered region of p27 was shown to cause a shift in its conformational landscape (Ban et al., 2017) stressing the capacity of IDRs as sensor of their environment (and not trivially, as drug targets).

Borrowing concepts from information theory (Shannon and Weaver, 1949), the capacity to transfer information is determined by the signaling event rate, and the size of the set formed by possible events it permits. Fuzzy complexes provide fast interconversion dynamics and a large set of configurations in the interface. Thus, fuzzy interfaces have the ability to act as high-capacity channels.

## Fuzzy interactions in Src family kinases

The Src N-terminal regulatory element (SNRE) studied by our group suggests an additional class of IDR allostery, in which the disordered region acts a sensor but the connecting element is a folded SH3 domain.

**Figure 2:**
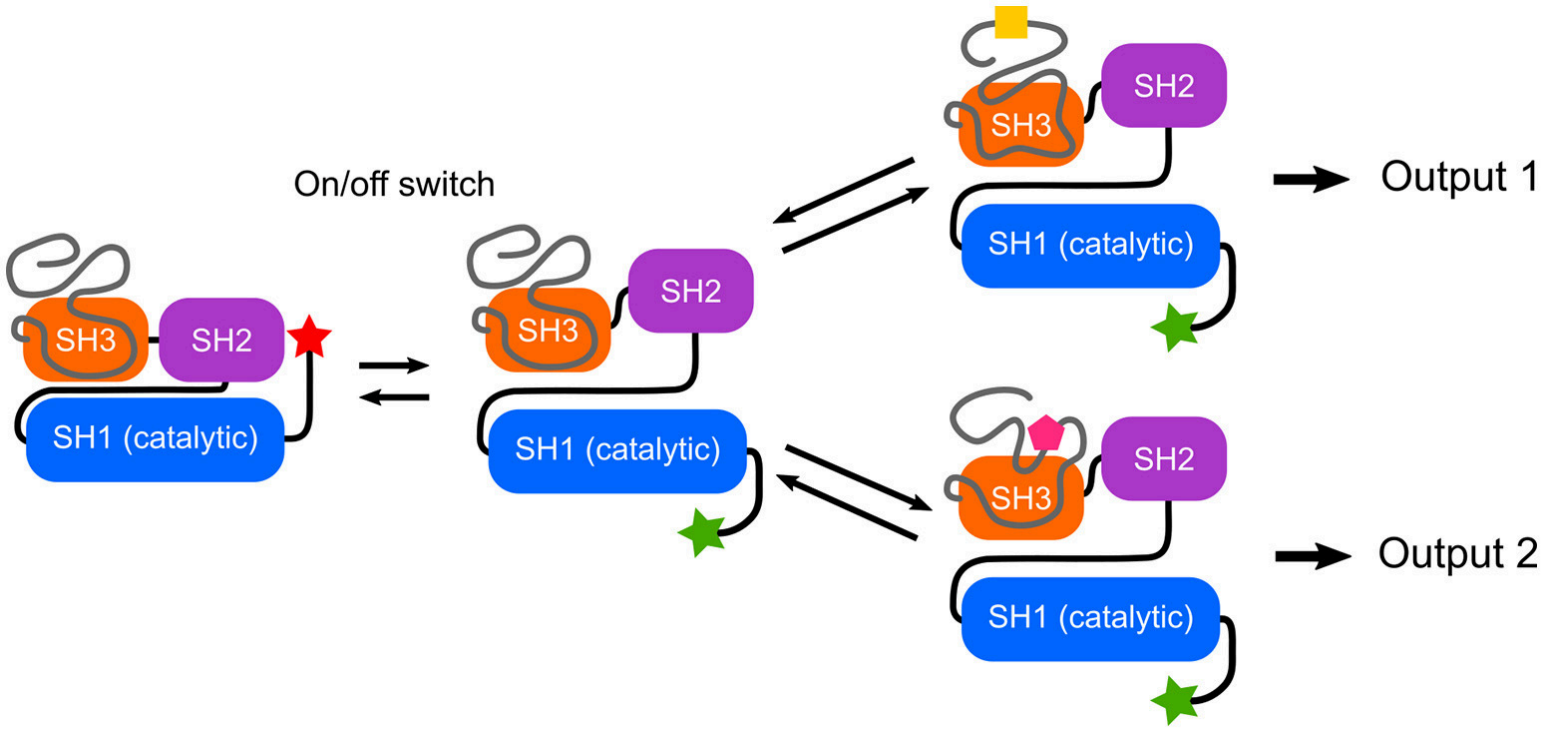
Switches and rheostats in Src Family Kinases. The conserved SH3-SH2-SH1 multidomain cassette implements an on/off switch based on the interaction between the phosphorylated C-terminal tail and the SH2 domain, as well as interactions involving the SH3 domain. The intrinsically disordered N-terminal region forms a fuzzy complex with the SH3 domain and enables a program that could direct different qualitative and quantitative outputs depending on the environment. Thus, the IDRs can function as selective rheostats. Within the Src Family of Kinases, the high homology among of the SH3-SH2-SH1 cassette contrasts with the uniqueness of the disordered N-terminal regions, suggesting a modular architecture in which specific responses are programmed in the IDR.

Mutations in the Unique domain of c-Src induce strong phenotypes in Src-dependent colorectal cancer cells (Arbesú et al., 2017). The Unique domain participates in a number of interactions with proteins such as calmodulin (Pérez et al., 2013) or N-methylaspartate receptor (Gingrich et al., 2004), lipids (Pérez et al., 2013) and is subjected to phosphorylation (Amata et al., 2014) and proteolytic processing (Hossain et al., 2013). In order to integrate these capabilities into a functional sensor-activator pair, the nature of the connector becomes a key issue. The SH3 domain has been shown to act as a scaffold of a fuzzy intramolecular complex (Maffei et al., 2015; Arbesú et al., 2017). These findings suggests that the SH3 domain may have a dual role in c-Src regulation: the traditionally recognized one, as a sensor (docking site) of polyproline peptide motifs, as well as that of a connector, relaying the information sensed by the preceding IDR (Figure 2). An enhanced capacity of SH3 motifs to interact with intrinsically disordered regions has been suggested (Beltrao and Serrano, 2005). Recently, the N-terminal IDR of Abl kinase has been shown to modulate its activity through the SH3 domain (Saleh et al., 2017).

Recent NMR data (Tong et al., 2017) as well as SAXS studies (Bernadó et al., 2008) confirm that the interaction between the SH3-SH2 regulatory domains and the kinase (SH1) domain is conserved in the active form of cSrc, i.e., in the absence of the autoinhibitory interaction between pTyr527 and the SH2 domain. The NMR results show that this interaction is dynamic and suggests that modulation of the interdomain dynamics may contribute to modulate c-Src activity.

In spite of their large sequence divergence, the IDR regions of the various SFK show coevolution with their respective SH3 domains, suggesting that a fuzzy interaction such as the one found in c-Src may be a functional element in all SFKs (Arbesú et al., 2017). The large sequence variations in the Unique domains contrasts with the very high homology displayed by the SH3-SH2-SH1 cassette, suggesting that the Unique domain has evolved to read the distinct environments required by each SFK.

The ΔPRE method is a simple and robust analytical approach to generate a fingerprint of the long-range interactions within intrinsically disordered domains. The complete processing tools are part of the Farseer software (Teixeira et al., in press).

Simulations based on fuzzy logic recapitulate many features of a kinase network (Aldridge et al., 2009). Proteins like the SFKs can be considered as algorithms reading complex signaling inputs to generate the proper responses. Thus, fuzzy interactions by IDRs may, in fact, be implementing fuzzy logic at the level of individual proteins.

## Author contributions

This is a mini-review article based on the Ph.D. thesis of MA, supervised by MP and with contributions from GI, HF, and JT working in fuzzy complexes of Src Family Kinases.

### Conflict of interest statement

The authors declare that the research was conducted in the absence of any commercial or financial relationships that could be construed as a potential conflict of interest.

The reviewer AJDQ and handling Editor declared their shared affiliation.
